# Thioredoxin System Regulation in the Central Nervous System: Experimental Models and Clinical Evidence

**DOI:** 10.1155/2014/590808

**Published:** 2014-02-27

**Authors:** Daniela Silva-Adaya, María E. Gonsebatt, Jorge Guevara

**Affiliations:** ^1^Laboratorio Experimental de Enfermedades Neurodegenerativas, Instituto Nacional de Neurología y Neurocirugía, 14269 México City, DF, Mexico; ^2^Departamento de Medicina Genómica y Toxicología Ambiental, Instituto de Investigaciones Biomédicas, Universidad Nacional Autónoma de México, 04510 México City, DF, Mexico; ^3^Departamento de Bioquímica, Facultad de Medicina, Universidad Nacional Autónoma de México, 04510 México City, DF, Mexico

## Abstract

The reactive oxygen species produced continuously during oxidative metabolism are generated at very high rates in the brain. Therefore, defending against oxidative stress is an essential task within the brain. An important cellular system against oxidative stress is the thioredoxin system (TS). TS is composed of thioredoxin, thioredoxin reductase, and NADPH. This review focuses on the evidence gathered in recent investigations into the central nervous system, specifically the different brain regions in which the TS is expressed. Furthermore, we address the conditions that modulate the thioredoxin system in both, animal models and the postmortem brains of human patients associated with the most common neurodegenerative disorders, in which the thioredoxin system could play an important part.

## 1. Introduction

The thioredoxin system (TS) consists of an electron donor and two types of antioxidant oxidoreductase proteins: thioredoxin (Trx) and thioredoxin reductase (TrxR) and NADPH as the electron donor. Trx was first identified as a hydrogen donor for ribonucleotide reductase in *Escherichia coli *[1]. Trx is a small 12 kD protein that has an active conserved site, Cys-Pro-Gly-Cys, which is essential for its function as both an active oxidoreductase and an electron donor of some peroxiredoxins that are important molecules for the reduction of peroxides [2]. Trx is also a regulator of cellular functions in response to redox signals and stress, modulating various signaling pathways, transcription factors, and immunological responses [3]. Trx is an important regulator of redox balance in the cell and has been implicated as playing a role in cell survival in many conditions including cancer and neurodegenerative diseases [4]. Human cells contain 3 different thioredoxins [5]. Trx1 has been reported as cytoplasmic, Trx2 as a mitochondrial form, and a third variant highly expressed in spermatozoa. Trx1 has been located in several cell compartments such as the nucleus and the plasma membrane or as a secreted protein [6, 7]. Posttranslational modifications to cysteine on Trx1 appear critical to its localization and function in different cell types [7]. Organelles such as the mitochondrion and nucleus require Trx to preserve a local reducing environment to minimize damage from ROS leakage during mitochondrial respiration [8]. In the nucleus the activation of transcription factor requires the presence of reduced Trx [9]. Cytosolic Trx1 is important in the control of growth and apoptosis and during chronic inflammation; likewise Trx1 is also secreted as a cocytokine and for chemokine activities [5].

TrxR is a homodimer, first described in bovine tissue by Holmgren and Luthman in 1978 [10], which catalyzes the reduction of the disulfide at the Trx active site, using NADPH with one FAD cofactor per subunit and a selenocysteine active site [5, 8]. There are three distinct genes in mammals that encode three different TrxRs: the cytosolic TrxR (TrxR1), mitochondrial TrxR (TrxR2), and thioredoxin-glutaredoxin reductase (TGR o TrxR3). TrxR1 and TrxR2 are expressed in all mammalian cells and tissues, while TrxR3 is expressed in the testicles [11].

Besides Trx, TrxR can directly reduce the number of other substrates, such as peroxides (including lipid hydroperoxides), hydrogen peroxide [12, 13], and protein disulfide isomerases, which participate in the posttranslational folding and processing of cellular proteins [14]. TrxR also participates in the regeneration of some antioxidant molecules with antioxidant activity such as dehydroascorbate [15, 16], lipoic acid [17], and ubiquinone [18].

The brain is more susceptible to oxidative damage compared with other organs, due to several factors that promote the formation of reactive species: high oxygen consumption, high iron levels found in some brain regions, and high fat content of unsaturated fatty acids, accompanied by low levels and low activities of some antioxidant enzymes such as superoxide dismutase (SOD), catalase, and glutathione peroxidase (Gpx) [19]. Both Trx and TrxR are widely expressed in tissues and organs; their distribution seems to be tissue and cell specific [20], including the brain tissue in which Trx and TrxR are found.

This review discusses the expression of the TS in different brain regions and cells and the participation of the TS in neurotoxic insults and the variety of neurodegenerative disorders where oxidative stress plays a key role.

## 2. Protein or mRNA Expression of Trx and TrxR in the Nervous System

The identification and localization of Trx and TrxR in the different brain regions have been made possible mostly through the use of immunochemistry techniques using monoclonal and polyclonal antibodies and by *in situ* hybridization techniques. Differentiation between the different isoforms is not always mentioned in the reports (Table 1). However, Trx, probably Trx1, due to its cytoplasmic localization, has been detected in the human brain and that of several mammal species including the rat, gerbil, cow (a yearling calf, more precisely), and mouse [10, 21–24]. Trx and TrxR were first identified in the sciatic nerve of the rat, in which both proteins showed strong cytoplasmic immunoreactivity in the Schwann cells at the Ranvier nodes and neuronal cells [20, 21]. Studies in rats demonstrate high levels of Trx mRNA in regions with high energy demands and high activity that involves redox reactive metabolites including the substantia nigra and the subthalamic nucleus. According to the authors, this suggests that the TS participates in the maintenance of the redox homeostasis in these regions. At the same time, the C1 area of the hippocampal formation shows very small expression in contrast to CA2/CA3 and the dental gyrus of the hippocampus. These are regions in contact with peripheral blood such as the choroid plexus which expresses a significant quantity of Trx mRNA [25]. Godoy et al. (2011) reported immunoreactivity to Trx1 in the Purkinje cell layer of the rat, as well as the motor neurons of the spinal cord, ependymal cell layer, and the cells of the choroid plexus. In contrast with Trx1, TrxR1 was abundantly expressed in the glial cells of the cerebellar white matter. Trx2 (mitochondrial Trx) was detected in the axonal fibers of the cerebral cortex, striatum, cerebellar white matter, and spinal cord, while TrxR2 expression was pronounced in the cell bodies of neurons found in the Purkinje and molecular cell layers in the cerebellum [24]. Godoy also evaluated the expression of Trx1, Trx2, TrxR1, and TrxR2 in the mouse brain, assessing the presence of the protein semiquantitatively (see Table 1) [26]. The mRNA and protein localization of Trx2 and Trx1 differ in some regions such as the hippocampus, and it is proposed that posttranscriptional regulation of Trxs may occur in this region. Another important observation is that in the rat Trx1 and Trx2 expression occurs predominately in brain neurons [25, 27], while TrxR protein levels are higher in glial cells than in neurons in both rat and mice cell cultures [20, 28]. These findings suggest that the functional needs and requirements of the TS molecules are different in each type of cell [25, 27].

Immunoreactivity of rabbit antiserum against rat liver Trx is found in the epithelial cells and secreting cells of the rat choroid plexus [20]. A human Trx homologue, adult T leukemia-derived factor (ADF/TRX), has been found to be widely expressed in the central nervous system (CNS); this includes the subcommissural organs, ependymal, tanycytes, and endothelial cells, as well as in the neuronal cell bodies of gerbils, albeit weakly [22], and the white matter astrocytes and Schwann cells in the posterior root of the human brain [23]. A truncated Trx1, thioredoxin 80 (Trx80), is present in the human brain in an aggregated form, principally in neurons [29]. The main role described for Trx80 is to activate monocytes, cause proliferation, and secrete pro- and anti-inflammatory cytokines [30].

**Figure 1:**
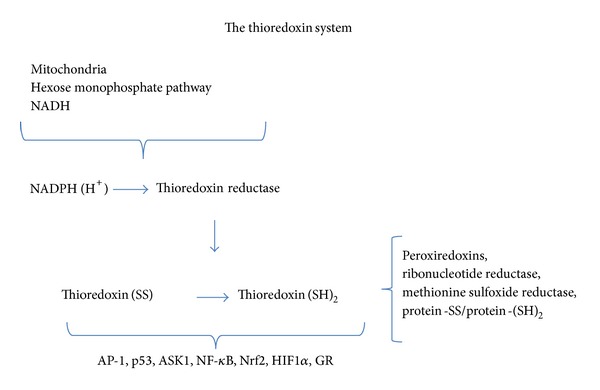
TS components are NADPH, TrxR, and Trx. NADPH is the electron donor for TrxR. Cytosolic NADPH generation principally occurs in the hexose monophosphate pathway, with mitochondrial NADPH production depending on specific dehydrogenases and the transference of electrons from NADH to NADP^+^ [35]. Trx acts as the reducing agent for peroxiredoxins, ribonucleotide reductase, methionine sulfoxide reductase, and disulfides in proteins including activating protein 1 (AP-1), tumour suppressor p53, apoptosis signal-regulating kinase-1 (ASK1), nuclear factor erythroid 2-related factor 2 (Nrf2), hypoxia inducible factor 1*α* (HIF1*α*), nuclear factor *κ*B (NF-*κ*B), and glucocorticoid receptor (GR) [36].

Despite its function as an intracellular disulfide reducing protein, Trx has been found in extracellular components, secreted mainly by activated T and B lymphocytes [31]. Studies have established that different forms of stimulation can cause different cells including nerve and glial cells to secrete Trx [32]. TrxmRNA has been localized also in epithelial cells of the choroid plexus and the ependymal cells of the ventricle and the secreting cells of the choroid plexus [25, 28]. The secretion of Trx into the cerebrospinal fluid may help protect nerve cells from oxidation by environmental influences maintaining a protective microenvironment [25]. Stemme et al. (1985) [21] and LoPachin and Barber (2006) [33] showed anterograde and retrograde axoplasmic transport of Trx and TrxR in stressed rat sciatic nerve cells; this transport to synaptic terminals may be involved in thiol redox reactions related to synaptic transmission, such as membrane pore formation by the participation of specific cysteine residues that modulate regulatory proteins. These observations are indirectly supported by the evidence showing the high sensitivity of the synaptic process to modifying nucleophilic sulfhydryl groups with different electrophilic neurotoxicants. Astrocytoma cells exposed to H_2_O_2_ release Trx1 into the culture medium. The addition of this medium to neuron cultures promotes their survival in the absence of serum [34]. These observations support the view that glial cells provide neurotrophic and antioxidant support for the neurons. The levels of Trx that are secreted by cells depend on their stage of metabolic activity (Figure 1) [25].

## 3. TS Modulation by Stress and Chemical Compounds

Trx expression is induced by stress, such as that produced by infectious agents, UV radiation, or O_2_ [37]. Furthermore, also many physicochemical agents and stimuli induce Trx gene expression, including hormones and nontoxic agents. CNS studies report a close association between the increased expression of Trx and TrxR and cell damage where oxidative stress is implicated. For example TS is upregulated in postmortem examination of AD where also oxidative stress has been documented [23, 38]. Mechanical nerve injury, such as sciatic nerve crush, induces TS components in rats [21]; as well middle cerebral artery occlusion [39, 40], hypoglossal nerve axotomy [41], and transient focal ischemia do so in both rats and gerbils [22, 42]. Exposure to several toxic chemicals that induce oxidative stress upregulates TS proteins. Enhanced immunoreactivity to Trx in the hippocampus and striatum is induced when rats are exposed to 3-nitropropionic acid, a mitochondrial complex II toxin [43]. The environmental pollutant formaldehyde has toxic effects on the CNS [44]. Trx1 expression increases in PC12 cells exposed to formaldehyde. This upregulation decreases if the exposure time increases [45]. Morphine, the most effective opioid analgesic, has pharmacological effects associated with cellular redox state [46]. SH-SY5Y cells exposed to morphine show augmented expression of Trx1, activating the opioid receptor and the phosphatidylinositol 3-kinase (PI3K) and extracellular signal-regulated kinases (ERK) signaling pathways [47]. Trx expression is also induced without stressor components such as compounds present in dietary intake; for example, fish oil increases the activity of TrxR in rat brain [48]. While t-bhq (t-butyl hydroquinone) increases the expression and activity of TrxR1 and TrxR2 in astrocytes, it does not in neurons [49]. Also, 17-*β* estradiol induces Trx protein expression in SH-SYE5Y cells, while the estrogen receptor activation is ligated to the upregulation of cytoprotective genes, including Trx via a cyclic guanosine monophosphate (cGMP) mediated signaling pathway [50].

Other studies have reported a downregulation or inhibition of the TS proteins. The effect may depend on the exposure time, dose, or the nature of the compound. Mice exposed to different concentrations of arsenic for four months showed that diminished Trx1 mRNA levels were in male striatum and the female nucleus accumbens [51]. Tellurium is present in optic and electronic technology, in batteries, and as an environment contaminant [52]. Diphenyl telluride induced prominent effects in mouse brain, including decreased TrxR activity [53]. Mercury compounds are accumulated in seafood and fish and readily cross the blood brain barrier [54]. Exposure to mercury compounds reduced TrxR activity in zebra fish brains, causing neurotoxicity through oxidative stress in this target organ and other organs such as the kidney [55]. The upregulation of the TS in response to different stressors is associated with neuronal survival mechanisms, which can protect against cell or tissue damage, while the inhibition or downregulation of TS leads to increased damage and cell death.

The promoter region for the constitutive expression of Trx1 contains various transcription factor binding sites, such as transcription factor SP1, GC-rich sequence DNA-binding factor (GCF), and wild type zinc finger (WT-ZFP), while the promoter regions for the inducible expression binding sites are AP-1, activating protein 2 (AP-2), NF-*κ*B, octamer binding transcription factor (Oct-1), polyoma enhancer activator 3 (PEA-3), myeloblastosis transcription factor (Myb), and the antioxidant-responsive element (ARE) [56]. The augmented expression of Trx1 and the activation of Nrf2 were observed in the peri-infarct regions of rats after middle artery occlusion [57] and prevented light-induced photoreceptor degeneration [58]. The presence of ARE is required for the induction of Trx1 in SH-SY5Y cells after hemin exposure and requires Nrf2 nuclear translocation downstream PI3K [59].

## 4. TS and Neuroprotection

Trx induction contributes to brain tolerance for and protection from toxic insults. Rats treated with selenium after receiving quinolinic acid treatment, a potent neurotoxin, show increased levels of protein and increased activity of TrxR1, ameliorating quinolinic acid damage [60]. Pretreatment with beta estradiol 3-benzoate ameliorates the injury induced by ferrous citrate in female rat brain. This protective effect is accompanied by increased Trx levels and activity [61]. The use of electroacupuncture produces clinically beneficial effects in stroke patients [62] and induces Trx expression in ischemic-reperfused rat brain [63]. Selegiline improves behavioral and cognitive functions in AD and PD. Selegiline protects SH-SY5Y cells against MPP^+^-induced neurotoxicity through the induction of the Trx gene via protein kinase A mediated by mitogen-activated protein kinases/extracellular signal regulated protein kinase 1/2 (MAP Erk1/2) and protooncogene protein c-Myc [64]. Microtubule associated protein-2 (MAP-2) and Tau protein are important in promoting and maintaining the neuronal cytoskeleton [65]. NOO^−^ and H_2_O_2_ induce thiol oxidation and disulfide formation in Tau and MAP-2, thus altering the ability of proteins to improve the assembly of microtubules *in vitro* from purified porcine tubulin. Treatment with TrxR restores the ability of MAPs to promote microtubule assembly [66]. Trx2 prevents the neurotoxicity that results from protein misfolding, due to the protein refolding effect of Trx on scrambled (mispaired disulfide-containing) RNase A and protein disulfide isomerase activity [67, 68].

Other experimental approaches enhanced the levels of Trxs expression *in vivo* and *in vitro*. Transgenic mice that overexpress human Trxs show enhanced levels of this protein in the brain. These mice show attenuated focal cerebral ischemic damage [40], seizures, and excitotoxicity induced by kainate [69], delayed retinal neurodegeneration in Tubby mice (a mouse model for retinal degeneration and loss of visual function). Tubby mice protection ocurred via Akt survival signal pathways and by increasing both the brain-derived neurotrophic factor (BDNF) and the glial cell line-derived neurotrophic factor (GDNF). In this experimental model, Trx overexpression inhibits the ASK1/JNK pathway [70, 71]. The overexpression or administration of human recombinant Trx (rTrx) on PC12 cells attenuates MPP^+^ neurotoxicity [72, 73]. Overexpression of human Trx1 and Trx2 protects retinal ganglion cells against oxidative stress-induced neurodegeneration [74]. Trx2 human overexpression in SH-SY5Y neuroblastoma cells prevents apoptosis and loss of the mitochondrial membrane potential induced by tert-butyl hydroperoxide [75]. The use of human rTrx has a protective effect in which the generation of reactive oxygen species (ROS) is involved in cytotoxic mechanisms [76]. Exogenously administered human rTrx ameliorates neuronal damage after transient middle cerebral artery occlusion in mice [42], reduces oxidative/nitrative stress and neuronal apoptosis after cerebral ischemia/reperfusion injury in mice [77], and augments neurogenesis following brain ischemia/reperfusion (I/R) injury in rats [78]. Studies *in vitro* demonstrate that the administration of rTrx increased neuronal cell survival in murine primary cultured neurons [34].

Other studies have described the preconditioning mechanisms as neuroprotection strategies that induce TS proteins and other antioxidant proteins. *In vivo* studies, in rats, show that hypobaric hypoxia preconditioning enhances Trx1 and Trx2 protein expression [79, 80]. *In vitro* studies show that the transient serum depletion of SH-SY5Y cells produces a hormetic response increasing Trx1 levels [81], which contributes to neuronal tolerance and protection against a posterior oxidative stress exposure. This type of Trx induction belongs to adaptive group of cytoprotective responses, allowing potentially recurrent stressors the survival to potentially recurrent stressors.

Mitochondrion is considered an important source of ROS, and the antioxidants systems play a significant role in this organelle. The two major scavenging systems in this organelle are GSH and Trx2. Trx2, together with GSH, plays an important role in the detoxification of H_2_O_2_ in the mitochondria of different types of brain cells in the rat hippocampus, to a greater extent even than other enzymes such as catalase [82]. Cellular GSH concentration ranges from ~2 to 10 mM depending on cell type in different species [19, 83], while Trx (isoform not specified) baboon tissues concentrations tend to be around ≤10 *μ*M, specifically in the brain 381 ± 110 pg/mg of protein [84]. Trx2 plays an important role in reducing other antioxidants, including peroxiredoxin 3 (Prx3), which is an antioxidant enzyme found exclusively in mitochondria [85]. Changes in Trx2 and Prx3 expression in the gerbil hippocampus after ischemic reperfusion may be associated with delayed neuronal death. The administration of Prx3 and Trx2 in ischemic brains shows substantial neuroprotective effects that reduce the oxidative stress induced by ischemia [86]. Trx2 plays an important role in the control of oxidative stress in mitochondria. Neurons with mitochondrial dysfunction (complex IV inhibition) show low levels of Trx mRNA and protein and are thus more vulnerable to H_2_O_2_. This vulnerability could be associated with the downregulation in the TS [87].

## 5. TS in Neuronal Development and Protection

Trx-2 and Trx-1 knockout mice present early embryonic lethality. Trx (isoform not specified) knockout mice embryos die shortly after implantation, and the concepti were resorbed prior to gastrulation, due a failed proliferation [88]. Studies *in vivo* and *in vitro*, deficient in Trx2, display increased cellular ROS, apoptosis, exencephaly, and early embryonic lethality [89, 90]. This evidence demonstrates that both Trx isoforms have essential roles in neuronal differentiation, proliferation, and survival. TS maintains a reductive environment in cells. Trx not only works as an antioxidant but also has other key biological activities, including growth control and antiapoptotic functions [91].

The nerve growth factor (NGF) is a neurotrophic factor playing an essential role in the development and promotion of survival and function of the CNS [92]. Likewise, Trx has protective effects that enhance the action of nerve growth factor via the regulation of antiapoptotic signaling and Trx's antioxidant activity. NGF induces Trx mRNA and protein levels via cyclic AMP responsive element (CREB) as well the nuclear translocation of Trx. The overexpression of the dominant negative type of Trx expression vector resulted in suppression of NGF-induced neurite outgrowth in PC12 cells, playing a critical regulatory role in NGF-mediated signaling transduction and outgrowth in PC12 cells [93, 94], via ERK [95]. Thus, Trx is a neurotrophic cofactor that augments the effect of NGF on neuronal differentiation and regeneration, showing neurotrophic activity in cholinergic neurons [96]. Trx is beneficial in cases of neurodegenerative disease, promoting neural-cell growth and aiding recovery [94].

Mechanisms by which thioredoxin regulates cell growth include binding to signaling molecules such as ASK-1 and thioredoxin-interacting protein (Txnip). ASK1 activates the c-Jun N terminal kinase (JNK) and p38 MAP kinase pathways and requires tumor necrosis factor (TNF-*α*) to induce apoptosis. Reduced Trx prevents apoptosis via an inhibitory binding to ASK1, which is lost when Trx is oxidized, which is mentioned later in this review. Trxip is an endogenous regulator of Trx that, with high affinity, binds to Trx and inhibits its ability to reduce sulfhydryl groups via NADPH oxidation and reduces the binding of Trx with ASK1 promoting an ASK1 apoptosis mediated pathway [97]. Evidence has established Trxip as a potent metabolic control protein [98, 99]. Several studies describe the control of the expression of Trxip during different conditions in the brain. Diabetic rat brains showed enhanced levels of Trxip mRNA, while Trx1 protein expression is enhanced after exercise in normal rats but not in diabetic rats [100, 101]. Trxip protein expression is induced in hyperglycemic-ischemic mice brains after middle cerebral artery occlusion [102]. Intravitreal NMDA injection augmented the expression of Trxip in rats, which was accompanied by both the release of inflammatory mediators TNF*α* and interleukin-1*β* (IL-1*β*) via ASK1 and the activation of the proapoptotic p38 MAPK/JNK pathway [103]. Exposure to silver nanoparticles induces the expression of the Trxip gene in different regions of the mouse brain [104]. The activation of ASK1 and the increase of Trxip levels produce apoptosis and neurotoxicity, making the cell more vulnerable to death. Hardingham and Bading [105] demonstrate that synaptic NMDAR activity inactivates Trxip via Forkhead box protein O (FOXO) transcription factor, enhancing Trx activity. NMDA receptor overactivity named “excitotoxicity” increased Ca^2+^ uptake by the mitochondria inducing ROS production. Thus Trx enhanced activity by NMDA receptor activity could reduce cell vulnerability to oxidative damage [105–107].

## 6. TS and Alzheimer's Disease

A common feature of Alzheimer's Disease (AD), Parkinson's Disease (PD), and Amyotrophic Lateral Sclerosis (ALS) is the extensive evidence of oxidative stress, which might be responsible for the dysfunction and death of neuronal cells that contribute to the pathogenesis of these diseases [108].

AD is the most common form of adult onset dementia. It is characterized by the presence of interneuronal filamentous inclusions, known as neurofibrillary tangles (NFT), and extracellular senile plaques (SP). Hyperphosphorylated Tau is the major protein involved in NFT. Amyloid beta peptide (A*β*), derived from the amyloid precursor protein, is the major protein in SP and amyloid angiopathy [109].

There is direct evidence that supports the theory of increased oxidative stress in the AD brain: (1) increased brain mercury, iron, and aluminum, capable of stimulating free radical generation, (2) increased lipid peroxidation, (3) increased protein and DNA oxidation, (4) diminished energy metabolism and decreased cytochrome c oxidase, (5) advanced glycation end products, malondialdehyde, carbonyls, peroxynitrite, heme oxygenase 1, and SOD-1 in NFT and SP, and (6) A*β* capability to generate free radicals. Overall, evidence suggests that free radicals are possibly involved in the pathogenesis of neuron death in AD and that the antioxidant systems could have an important role in the prevention and control of AD [110].

In AD brains, the ADF/Trx expression in astrocytes of white matter increased (Table 2) [23], while it was found to decrease in some regions of AD brains, in comparison with the controls [38, 111]. Trx80 is also drastically decreased in AD brains. Trx80 inhibits A*β* aggregation and protects against its toxicity, reducing neuronal vulnerability [29]. In amnestic mild cognitive impairment (AMCI, a transition stage between normal aging and AD), brains examined postmortem were characterized by diminished Trx-1 levels in the hippocampus and cerebellum [112]. Rats exposed to chronic intermittent hypoxia exposure, a reversible cause of cognitive loss in patients with AD [113], show impaired spatial learning and memory that are negatively correlated with Trx protein and ARN levels in the hippocampus [114]. Nevertheless, TrxR activity was increased in the cerebellum and amygdala of AD brains, suggesting that TrxR activities increase, perhaps as a compensatory mechanism in the face of increased oxidative stress that is limited by the substrate Trx, and could contribute to the general increase in oxidative stress and subsequent neurodegeneration seen in AD [38].

*In vitro* studies demonstrated augmented levels of oxidized Trx1 and an increase in the levels of apoptosis in SH-SY5Y cells exposed to A*β* [111]. Reduced Trx is a negative regulator of apoptosis via ASK1 [115]. Studies also show that ASK1 participates in A*β* induced neuronal cell death [116]. The overexpression of Trx1 protects SH-SY5Y cells against A*β* [111]. Furthermore, Trx and TrxR treatments protect primary hippocampal cultures from A*β* toxicity, acting as radical scavenger that inhibits the neuronal injury induced by A*β* [38]. A*β* has a critical methionine residue at position 35 [117]. The reversible product of methionine oxidation is methionine sulfoxide and can be reduced by methionine sulfoxide reductases based on TrxR regulation, while the irreversible oxidation to methionine sulfone is rare and only takes place in the presence of strong oxidants [118]. Methionine sulfoxide modulates oxidative stress and the neurotoxic properties of A*β*, and methionine sulfoxide reductase activity is reduced in the AD brain [119]. A*β* is related to the pathogenesis of other disorders like macular degeneration and glaucoma in mice via the impairment of the TS [120]. A*β* modifications depend on the TS, and the diminished levels of this protein system make the cell more vulnerable to neurotoxic A*β*.

## 7. TS and Parkinson's Disease

Idiopathic PD is characterized clinically by tremor, rigidity, bradykinesia, and posture instability [121]. PD is diagnosed pathologically by the loss of neurons in the substantia nigra pars compacta of the midbrain, in association with the widespread occurrence of Lewy bodies (intracytoplasmic filamentous aggregates of *α*-synuclein present in neurons and axons) [122]. Oxidative stress is present in PD, probably due to factors such as increased iron levels, low GSH levels and the impairment of mitochondrial complex I function in the substantia nigra [123].

In patients with sporadic PD, oxidative forms of DJ-1 protein were found [124]. DJ-1 acts as an antioxidant and transcription factor, having been observed in studies as protecting the culture cells and substantia nigra of mice from oxidative stress by inducing Trx1 expression via the transcription factor Nrf2 [125]. Nrf2 transcription factor is related to the expression of antioxidant and detoxifying enzymes, including Trx and TrxR [126]. *α*-Synuclein inclusions are common in PD, where its methionines and tyrosines are susceptible to oxidation [127]. The oxidation of synuclein methionines stabilizes soluble oligomers, while hetero-oligomers composed of synuclein and oxidized synuclein could have a toxic impact on the cellular environment [128].

Paraquat, MPP^+^, and rotenone are chemical compounds that mimic PD in animals and exert their toxic actions through the inhibition of mitochondrial complex I, inducing dopaminergic degeneration, as found in rodents. These compounds have been used for the study of neurotoxic mechanisms in PD [129, 130]. Ramachandiran et al. (2007) reported that, in SK-DAT cells, a different mechanism of cell damage operates in the TS, in which paraquat oxidizes Trx1 while rotenone and MPP^+^ oxidize Trx2. Chen et al. (2010) reported that in PC12 cells exposed to MPP^+^ decreased expressions of Trx1 and Trx2, although MPP^+^ decreased the expressions of both Trxs, the ratio of oxidized versus reduced Trx1 and Trx2 was relatively increased. This could explain how each toxin works at different levels within the cell, with rotenone and MPP^+^ working at a mitochondrial level and Paraquat at a cytosol level in relation to the TS [131, 132]. Another CNS toxin used in PD animal models is the fungicide Maneb, which is a mitochondrial complex III inhibitor. Roede et al. (2011) probed Paraquat and Maneb in SH-SY5Y neuroblastoma cells, finding that Paraquat oxidizes Trx2, whereas Maneb induces the expression of TrxR1, which correlated with the abundant nucleus increase of the transcription factor Nrf2 [133]. Consistently, studies have shown that Trx also protects both SH-SY5Y and PC12 cells against the severe oxidative stress and damage caused by the parkinsonism-producing neurotoxin MPP^+^ [81, 85, 134]. In mice exposed to MPP^+^, the activation of both ASK1 and its downstream target JNK was observed, which implicates Trx in the ASK1-mediated redox signaling in the pathogenesis of PD [135, 136].

Mitochondria are the major source of ROS, which are implicated in the pathogenesis of neurodegenerative diseases such as PD [137]. The TS has a significant role in H_2_O_2_ detoxification and the consequent cell death in dopaminergic cells. In dopaminergic cells exposed to 6-hydroxydopamine and paraquat, the inhibition of TrxR, induced mitochondrial dysfunction, increased H_2_O_2_ levels and cell death through oxidative stress [138]. Studies of the nigral DA cell line after H_2_O_2_ using microarray analysis to identify several groups of genes regulated by oxidative stress and related to functional mitochondrial complex I molecules, exocytosis, membrane trafficking, and Trx1 [139].

## 8. Other Neurodegenerative Diseases

Huntington's disease (HD) is a neurodegenerative disorder, most of whose clinical features can be attributed to CNS neurodegeneration, with up to 95% loss of GABAergic neurons from striatum [140]. Oxidative stress has been proposed as either a causative event or as a secondary constituent of the cell death cascade in HD [141]. The reported reduction of Trx1 and TrxR1 in the plasma and erythrocytes in blood samples from HD patients [142] evidenced an oxidative stress peripheral response to this neurodegeneration. Schizophrenia has a range of cognitive deficits that may involve oxidative stress and possibly contribute to cognitive deficits during aging and in neurodegenerative disorders [143]. Various studies have shown increased levels of Trx in plasma and serum in first episode schizophrenia patients and enhanced Trx levels in the plasma of long-term schizophrenic patients [144–146].

Postmortem of spinal cords presenting amyotrophic lateral sclerosis (ALS) and the erythrocytes of familial amyotrophic lateral sclerosis (FALS) with stable forms of mutant SOD-1 proteins show that Trx genes and protein expression are upregulated [147, 148]. Both studies suggest the involvement of Trx in the etiology and progression of the disease.

## 9. Concluding Remarks

Evidence shows that the presence of TS proteins is differential in the brain. Since the activity of Trx and TrxR is related to the activation of genes, the cellular cycle, and, especially, cell protection and survival, this differential expression suggests that some brain regions have different requirements for TS proteins for cell functions or against ROS damage. We have revised the modulation of the TS in different animal models, discussing the various mechanisms activating the TS and the mechanisms through which it exercises its functions. These studies demonstrate that the upregulation of TS proteins is accompanied by cell protection against damage, while the downregulation makes cells more vulnerable to death. Research in postmortem brains from different neurodegenerative disorders shows a differential modulating pattern in these disorders. It may depend on disease's stage, which makes the TS a therapeutic target for the treatment and retardation of several neurodegenerative processes. The role on antioxidant functions is important but even more than the antioxidant activity; TS proteins by their redox properties modulate the function and expression of other proteins, including different transcription factors essential for the development and for the control of cell survival or death. Elucidation of specific functions and mechanism of regulation of TS is required in different brain cell types. The role of Trx secretion and the functions as a brain cocytokine and chemokine is needed as well; this will be helpful for the study in pathogenesis of different neurodegenerative diseases.

## Figures and Tables

**Table 1:** TS expression in different species and brain regions.

Species	Protein	Findings (localization/expression)	Detection method	Reference
Calf	Trx	↑ Kidney, liver, brain, thymus	Radio immunoassay, rabbit antiserum, calf liver Trx and ^125^I-labeled Trx	[10]

Rat sciatic nerve	Trx	↑ Cytoplasm of Schwann cells Nodes of Ranvier	Immunofluorescence with specific rabbit antisera.	[21]
	TrxR	↓ Axoplasm of myelinated axons		

Gerbil brain	ADF/Trx	↓ Ependyma, tanycytes Endotheliall cells Neuronal cell bodies	Immunochemisty anti-human ADF antibody	[22]

Rat brain	Trx	↑ Paraventricular hypothalamic Nucleus Locus coeruleus Nucleus of the solitary tract	*In situ* hybridization Human Trx mRNA	[25]
		↓ Frontoparietal cortex Caudate/putamen Magnocellular preoptic nucleus		

Human brain	ADF/Trx	White matter astrocytes Schwann cells of posterior root	Immunochemistry	[23]
		White matter astrocytes	*In situ* hybridization and semiquantitative mRNA	

Mouse	Trx1	Nucleus of granular cells in hippocampus Golgi cells of substantia nigra Purkinje cells Motor neurons of the spinal cord	Anti-mouse Trx1	[24]
	Trx2	Golgi cells of Substantia nigra Axonal staining in cerebral cortex Axons in striatum Axonal staining in cortex Axon-bundles in striatum Golgi cells of substantia nigra	Anti-human Trx2	
	TR1	Faint staining in hippocampus pyramidal cells	Anti-rat TrxR1	
	TR2	Golgi cells of substantia nigra Strong staining in Purkinje cells Molecullar layer in cerebellum	Anti-rabbit TrxR2 (Santa Cruz Bio. sc-67127)	

Rat brain	Trx1	↑ Cerebellum Cortex Substantia nigra Retinal Spinal cord ↓ Striatum hippocampus	Anti-mouse Trx1	[26]
	Trx2	↑ Striatum Substantia nigra ↓ Cerebellum Hippocampus Cortex, Retinal Spinal cord	Anti-human Trx2	
	TR1	↑ Cerebellum Hippocampus Striatum ↓ Cortex Substantia nigra Spinal cord Retina	Anti-rat TrxR1	
	TR2	↑ Cerebellum ↓ Striatum	Anti-rabbit TrxR2 (Santa Cruz Bio. Sc-67127)	

↑: high protein content; ↓: low protein content. The origin of the antibodies employed is mentioned when provided in the reference cited.

**Table 2:** TS and CNS disorders.

Disorder	Cell type studied	TS expression	Reference
AD	AD human brain	**↑** ADF/Trx (p, mRNA) astrocytes in white matter	[23]
		**↓** Trx1 (p) amygdala, hippocampus, and frontal cortex	[38, 111]
		**↓** Trx80 (p) neurons of hippocampus and cortex **↓** Trx80 (p) in CSF	[29]
	AMCI human brain	**↑** TR (a) hippocampus and cerebellum	[112]
	SH-SY5Y cells exposed to A*β*	**↑** Trx1 oxidized	[111]

PD	PC12 cells exposed to MPP^+^	**↓** Trx1 **↓** Trx2 **↑** Oxidized Trx1 Trx1-SS **↑** Oxidized Trx2-SS	[132]
	SK-DAT cells exposed to paraquat	Oxidizes Trx1	[131]
	SK-DAT cells exposed to rotenone and MPP^+^	Oxidizes Trx2	
	SH-SY5Y cells exposed to paraquat	Oxidizes Trx2	[133]
	SH-SY5Y cells exposed to Maneb	**↑** TR1 (mRNA)	

HD	HD patients	**↓** Trx plasma and erythrocytes **↓** TR plasma and erythrocytes	[142]

Schizophrenia	First episode psychosis Long-term schizophrenic	**↑** Trx serum or plasma levels **↑** Trx serum or plasma levels	[144–146]

ALS	Spinal cord of ALS	**↑** Trx (mRNA)	[148]

FALS	FALS erythrocytes stable form of mutant SOD-1	**↑** Trx (p)	[147]

(p): protein expression; (a): activity; **↑**: upregulation; **↓**: downregulation.
